# Stilbenes in *Carex acuta* and *Carex lepidocarpa*

**DOI:** 10.3390/molecules29163840

**Published:** 2024-08-13

**Authors:** Jan Tříska, Naděžda Vrchotová, Štěpán Horník, Jan Sýkora, Andrea Kučerová

**Affiliations:** 1 Laboratory of Metabolomics and Isotope Analyses, Global Change Research Institute, Czech Academy of Sciences, Bělidla 986/4a, 603 00 Brno, Czech Republic; vrchotova.n@czechglobe.cz; 2 Department of Environmental Engineering, Institute of Chemical Process Fundamentals, Czech Academy of Sciences, Rozvojová 2/135, 165 02 Prague, Czech Republic; hornik@icpf.cas.cz; 3 Department of Analytical Chemistry, University of Chemistry and Technology, Technická 5, 166 28 Prague, Czech Republic; jan1.sykora@vscht.cz; 4 Department of Experimental and Functional Morphology, Institute of Botany, Czech Academy of Sciences, Dukelská 135, 379 01 Třeboň, Czech Republic; andrea.trebon@seznam.cz

**Keywords:** *Carex acuta*, *Carex lepidocarpa*, pallidol, miyabenol A and C, *trans*-ɛ-viniferin, liquid chromatography, NMR

## Abstract

Stilbenes in the roots of *Carex acuta* and *Carex lepidocarpa* were studied. Root samples were extracted with 100% methanol and analyzed by HPLC and LC-MS. In this way, *trans*-resveratrol dimers (*m*/*z* 455 Da [M + H]^+^), trimers (*m*/*z* 681 Da [M + H]^+^) and tetramers (*m*/*z* 907 Da [M + H]^+^) were identified in the extracts. Using LC-NMR in stop-flow mode, pallidol and *trans*-ε-viniferin as dimers were identified. After the separation of individual peaks and their measurement by ^1^H NMR, *cis* and *trans*-miyabenol A as a tetramer and *cis*-miyabenol C as a trimer were identified. In the case of miyabenol A, it is a chromatographically inseparable mixture of *cis* and *trans* isomers in the ratio of 2:3 according to ^1^H NMR measurement. In the case of *cis*-miyabenol C, the *Z-trans-trans*-miyabenol C configuration was confirmed. The remaining unidentified peak with a practically identical UV-VIS spectrum to that of *cis*-miyabenol C is most likely another isomer of miyabenol C.

## 1. Introduction

The largest genera in the *Cyperaceae* family are *Carex* and *Cyperus.* The *Carex* genus is one of the largest angiosperm genera with worldwide distribution. The total number of *Carex* species in the world is estimated to be more than 2000; among those, 222 species occur in Europe [1].

*Carex acuta* L. 1753 (syn. *Carex gracilis*, Curtis 1782) occurs throughout Europe, from NW Africa to Central Asia. It is a relatively eurytopic species, tolerates strongly acidic to neutral soils (pH ca. 4–7) and eutrophication and grows at river banks, the shorelines of lakes and fishponds, and the margins of wet meadows, canals and fens [2].

*Carex lepidocarpa* Tausch 1834 (syn. *C. flava* var. *lepidocarpa* (Tausch) Godron 1844; *C. viridula* Michaux var. *lepidocarpa* (Tausch) B. Schmid 1983, *C. lepidocarpa* subsp. *lepidocarpa)* is a part of taxonomically problematic *C. flava* group. *C. lepidocarpa* was often treated under the polymorphic *C. viridula*, e.g., *C. viridula* subsp. *brachyrhyncha* (Schmid 1983); however, recent studies confirm *C. lepidocarpa* as a separate species [3,4], and it was referenced under this name in the recent European checklist of the *Carex* genus [1]. It occurs throughout Europe, NW Africa and E Canada. It is a relatively stenotypic species, is sensitive to changes in the water table and overgrowing and prefers base-rich substrates. It grows in mesotrophic minerotrophic peat bogs or fen meadows [2]. *C. acuta* usually develops large stands along the margins of standing waters or forms big tufts (up to 1 m high) in shallow water bodies and in mesotrophic wetlands. It has a high growth rate; therefore, it produces a relatively high amount of above- and belowground biomass. It is possible to grow it easily in a simple hydroponic setting at a low cost [5] and produce a sufficiently high amount of root biomass (root length up to 0.8 m in a shallow water body) for the next extraction of valuable biological compounds. In contrast, *C. lepidocarpa* forms smaller tufts (0.2–0.5 m high), probably has a lower growth rate and grows only sporadically under natural conditions. Nevertheless, it is possible to also grow it in a simple hydroponic setting, as this species can tolerate a high water table and a low content of nutrients. However, the total available root biomass will probably be lower than in *C. acuta*, as a significant relationship between above- and belowground biomass was reported for many herbs [6]. The production of root biomass and growth rate should be tested and evaluated in terms of space, time and the economic costs of production of these biologically interesting compounds. Sedges contain a wide range of stilbenes, mainly in the roots; the set of stilbenes among species differs considerably (Table 1). Stilbenes and their derivatives, especially *trans*-resveratrol oligomers, have attracted increasing attention for potential pharmacological applications due to their promising biological activities. The most promising application field for resveratrol oligomers from sedges is the treatment of cancer and Alzheimer’s disease. Resveratrol oligomers isolated from *Carex folliculata* and *Carex gynandra* (pallidol, α-viniferin, *trans*-miyabenol C and kobophenols A and B), along with resveratrol, were evaluated for antiproliferative effects against human colon cancer (HCT-116, HT-29, Caco-2) and normal human colon (CCD-18Co) cells. The mentioned resveratrol oligomers, as well as resveratrol, inhibited the growth of the human colon cancer cells, and the most active compound was found to be α-viniferin, with IC_50_ values of 6–32 μM [7]. Hu et al. [8] found that miyabenol C, isolated from the stems and leaves of the small-leaf grape (*Vitis thunbergii* var. *taiwaniana*), can inhibit both in vitro and in vivo β-secretase activity, which would lead to a reduction in the accumulation of amyloid-β-peptide in the brain as the primary cause of Alzheimer’s disease. Wang et al. [9] described very recently that miyabenol C and *trans*-ε-viniferin, two resveratrol oligomers, specifically inhibit SARS-CoV-2 entry by targeting host protease cathepsin L. This means that there is a potential application of these resveratrol oligomers present in *Carex* sp. as lead compounds in controlling SARS-CoV-2 infection.

We focused in our study on two species of sedges, *Carex acuta* and *Carex lepidocarpa*, first, due to the content of stilbenes, which has been not studied yet (see Table 1), and second, due to correlation on a nutrient gradient. *C. acuta* is more typical of eutrophic pond shores and eutrophic wetlands, while *C. lepidocarpa* is more typical of oligotrophic peat bogs or fen meadows. Another difference is that *C. acuta* tolerates large water table fluctuations, while *C. lepidocarpa* prefers a more balanced water regime.

Since we are also dealing with stilbenes in grape canes, our next goal was to compare the contents of the main biologically active stilbenes, with the focus of interest being trimers and tetramers of resveratrol, which are contained in grape canes in smaller amounts compared to those *Carex* species studied above, which were selected based not only on the above facts and literature data but also based on a quick screening in the wetland plants collection.

## 2. Results and Discussion

**Figure 1:**
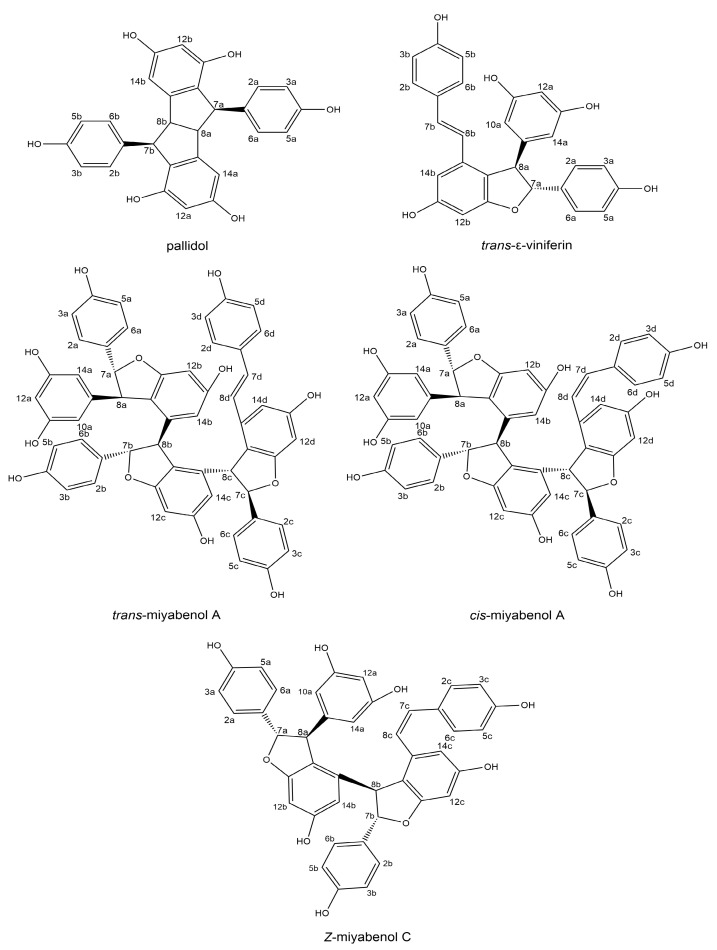
Chemical structure of some stilbenes.

**Figure 2:**
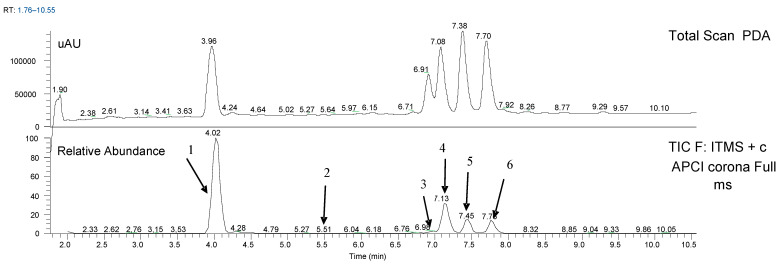
LC/MS of *Carex lepidocarpa* extract (PDA; full scan: +APCI). See Table 2 for the description of the peaks (1–6).

Recently, a very extensive review was published [21], in which the authors state that from *Carex* genera, 17 stilbenes have been isolated so far. *Carex acuta* and *Carex lepidocarpa* are not listed in this review. Another overview was published this year [22], but it does not change anything about the results listed above. From our literature review (see Table 1), it follows that in all *Carex* species, mostly in the roots but also in the seeds, the following resveratrol derivatives are repeatedly present: resveratrol diglucoside, piceatannol, α-viniferin, ɛ-viniferin, kobophenol A, kobophenol B, miyabenol A, B, C and pallidol. From this group, three substances are the most important due to the valuable biological properties described so far: miyabenol C, ɛ-viniferin and pallidol, and all of the mentioned stilbenes are contained in the *Carex* spp. The chemical structures of some stilbenes are shown in Figure 1, and a chromatogram of the LC/MS analysis of *C. lepidocarpa* extract is shown in Figure 2. We focused our study, in accordance with the indicated chromatogram, on the following stilbenes, except resveratrol: pallidol, a mixture of *cis*- and *trans*-miyabenol A, *trans*-ε-viniferin, *Z*-miyabenol C and peak 6. The molecular masses of the stilbenes were determined by LC/MS in positive mode and are listed in Table 2 together with the retention data. The content of stilbenes in *Carex acuta* and *Carex lepidocarpa* is shown in Table 3. Further detailed analysis was performed using LC/NMR.

The primary analysis of the stilbenes’ structures was based on LC-NMR, which provided spectra of individual peaks. Due to the low concentrations of individual compounds, only ^1^H NMR spectra in stop-flow mode were recorded within a reasonable time. This led to the identification of pallidol and *trans*-ε-viniferin as peak 1 and peak 4 from *C. lepidocarpa*, respectively. The identification of pallidol was achieved by a comparison of the measured ^1^H NMR spectrum with the literature data [23], whereas the spectrum of the compound from peak 4 was identical with a previously measured spectrum of *trans-*ε-viniferin [24], which served as proof for the compound identity.

The spectra of the rest of the compounds were more complex and could not be identified directly from LC-NMR. Therefore, the individual peaks were collected in repeated HPLC runs, evaporated and dissolved in corresponding deuterated solvents (acetone-*d_6_*, methanol-*d_4_*) for a comparison with the available literature data. The LC-MS analysis revealed that peak 5 and peak 6 from *C. lepidocarpa* are resveratrol trimers, while peak 3 from *C. acuta* is a resveratrol tetramer.

**Figure 3:**
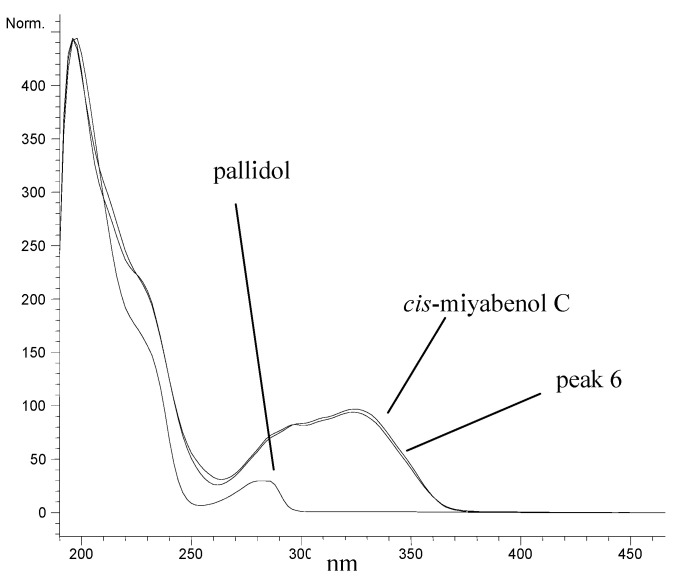
UV spectrum of selected peaks.

The comparison with the available literature [25] showed that peak 5 from *C. lepidocarpa* was represented by *Z*-miyabenol C (*cis*-miyabenol C). This compound is characterized by a *Z*-conformation on the double bond. This is evident from the signals at δ 5.83 ppm and 5.78 ppm, which are coupled with a value of the corresponding coupling constant (*J* = 12.4 Hz) characteristic for *Z*-conformation. Both benzodihydrofuran rings (first, δ 5.29 ppm and 4.22 ppm; second, δ 5.26 ppm and 3.87 ppm) have *trans* relative stereochemistry according to the literature data [25]. Therefore, this compound is *Z*-*trans*-*trans*-miyabenol C. Unfortunately, no match was found for peak 6 from *C. lepidocarpa*, and the compound structure could not be elucidated unequivocally. However, we believe that this compound might be an *E*-isomer of miyabenol C. This belief comes from a tentative analysis of ^1^H NMR and COSY spectra, which showed signals of a double bond with *E*-conformation (δ 6.89 and 6.26 ppm with *J* = 16.3 Hz). Moreover, the compound is probably represented by two benzodihydrofuran rings (δ 5.30 and 3.70 ppm, coupled with *J* = 3.6 Hz, and δ 5.03 and 4.58 ppm, coupled with *J* = 7.3 Hz, for protons on the dihydrofuran parts of the rings and δ 6.30 and 5.97 ppm, coupled with *J* = 2.2 Hz, and δ 6.73 and 6.20 ppm, coupled with *J* = 2.1 Hz for protons on the benzo parts of the rings). Besides that, there is a 3,5-dihydroxyphenyl ring with signals at δ 6.22 and 5.97 ppm coupled with *J* = 2.3 Hz and three 4-hydroxyphenyl rings (first, δ 7.07 and 6.81 ppm, coupled with *J* = 8.6 Hz; second, δ 6.99 and 6.74 ppm, coupled with *J* = 8.6 Hz; and third, δ 7.00 and 6.80 ppm, coupled with *J* = 8.7 Hz). Rotation of the third 4-hydroxyphenyl ring might be slightly restricted, which is assumed based on the width of its signals. Unfortunately, we have no other proof for this theory. To the best of our knowledge, this proposed isomer of *E*-miyabenol C has not been identified before. Nevertheless, it is impossible to determine the stereochemistry without further experiments, for which we have too small an amount of the compound. The UV-VIS spectrum of the compound represented by peak 6 is shown in Figure 3, where it is compared with the measured spectrum of *cis*-miyabenol C, which was already published by [14]. It is obvious that according to the UV-VIS spectra, the structure of the unknown compound (peak 6) must be very close to that of *cis*-miyabenol C. The UV-VIS spectrum of pallidol is also shown for comparison.

The ^1^H NMR spectrum of Peak 3 revealed that this peak is most likely represented by a mixture of the *trans*- and *cis*-isomers of miyabenol A, as this spectrum was compared to the literature data [11,18]. The ratio of *trans–cis* isomers is 3:2 according to the ^1^H NMR experiment. Nevertheless, the strongly overlapped signals could not be distinguished, and the assignment is based on isolated signals and signals of hydrogens bonded to double-bond carbons. To confirm the elucidation, the solution of Peak 3 was left exposed for a week at solar radiation, which lead to the transformation of the *trans*-isomer to the *cis*-isomer of miyabenol A. Similarly, to *Z*-miyabenol C, *cis*-miyabenol A is characterized by a *Z*-conformation on the double bond (δ 5.89 and 5.85 ppm, J = 12.0 Hz). Moreover, the relative stereochemistry on all three benzodihydrofuran rings is *trans* according to the literature data [18].

All of the above-mentioned stilbenes are contained in both *C. acuta and C. lepidocarpa*, but in different proportions. In *C. acuta*, a mixture of *cis* and *trans* miyabenol A, which is a tetramer of resveratrol, predominates, *trans-*ɛ-viniferin is not present at all, and the concentrations of other stilbenes vary in the range of hundreds of μg/g d.m. In *C. lepidocarpa*, the situation is the opposite. If we do not count *trans*-resveratrol, the concentration of which varies in both species in tens of μg/g d.m., then the most represented substance is pallidol, up to 14.48 mg/g d.m. The content of other substances is lower and quite balanced, e.g., *trans*-ɛ-viniferin in a concentration of 3.78–5.04 mg/g d.m., *cis*-miyabenol C in a concentration of 3.21–4.81 mg/g d.m. and peak 6 at a concentration of 4.07–5.06 mg/g d.m. If we summarize the contents of stilbenes in *C. lepidocarpa* dried in 2016 at lab. temperature, we reach a relatively high value of 31.28 mg/g d.m. for the total stilbenes content. The amount about of 3% *w*/*w* becomes interesting for the isolation of these substances from the biomass of *C. lepidocarpa* roots.

Quantitative data on the content of stilbenes are relatively rare in the literature. Suzuki at al. [14] state that the total content of stilbenes in the roots and rhizome of *C. fedia* var. miyabei is over 0.15% (*w*/*w* f.w.). The amount of kobophenol B in *C. gynandra*, *C. pendula* and *C. pumila* ranges between 0.1 and 1.27% *w*/*w* d.m. [7,18] which means that our results of the stilbene content in *C. lepidocarpa* show the highest stilbene content reported so far.

## 3. Materials and Methods

### 3.1. Plant Materials

Roots of *Carex acuta* (IPEN nr. CZ 0 HBT 2017.03812) and *Carex lepidocarpa* (IPEN nr. CZ 0 HBT 2017.03753) were provided by the Collection of Aquatic and Wetlands Plants in Třeboň, which is a part of the Institute of Botany of the Czech Academy of Sciences, Czech Republic. The plant materials were dried at room temperature; in 2017, a part of the material was dried at room temperature and another part was freeze dried.

The dried material was crushed and extracted with 100% methanol at 50 °C for 3 h. After centrifugation, the sediment was washed twice more with methanol. Three parallel samples were always prepared. Until measurement, the extracts were stored at −20 °C.

### 3.2. Chemicals

Methanol LiChrosolv gradient grade for LC (Merck, Prague, Czech Republic), acetonitrile Optima LC/MS (Fisher Scientific, Pardubice, Czech Republic), *ortho*-phosphoric acid, p.a. (Fluka, Prague, Czech Republic) and formic acid (Merck, Prague, Czech Republic) were used. Standards of *trans*-resveratrol and *trans*-ε-viniferin were purchased from Merck (Prague, Czech Republic).

### 3.3. HPLC and LC/MS

#### 3.3.1. HPLC

HPLC—the methods described in our previous publication [24] were used for HPLC. The samples were analyzed using an HP 1050 (Ti-series) HPLC instrument (Hewlett Packard, Palo Alto, CA, USA) on a 3 μm, 150 mm × 2 mm, Luna C18(2) column (Phenomenex, Torrance, CA, USA) with a water-acetonitrile-*o*-phosphoric acid mobile phase. Mobile phase A used 5% of acetonitrile + 0.1% of *o*-phosphoric acid; mobile phase B used 80% of acetonitrile + 0.1% of *o*-phosphoric acid (vol.%). The gradient was increased from 20% of B to 80% of B during 20 min and from 80% of B to 100% of B during 5 min. The flow rate was 0.250 mL/min and the column temperature was 25 °C. The injection volume was 5 μL. A diode array detector (G1315B DAD, Agilent, Prague, Czech Republic) with detection wavelengths at 220 and 315 nm and a scanning range of 190–600 nm was used, as well as a G1321A fluorescence detector (FLD, Agilent, Prague, Czech Republic) with an excitation wavelength of 315 nm, an emission wavelength of 395 nm and a scanning of emissions in the range of 300–600 nm. Quantification was performed according to the calibration curve, and the LOD and LOQ values are as follows: *trans*-resveratrol according to the calibration curve for *trans*-resveratrol at 315 nm (LOD 0.033 μg/mL, LOQ 0.109 μg/mL), pallidol according to the calibration curve for *trans*-resveratrol at 220 nm (LOD 0.056 μg/mL, LOQ 0.187 μg/mL), other stilbenes according to the calibration curve for *trans*-ɛ-viniferin at 315 nm (LOD 0.089 μg/mL, LOQ 0.298 μg/mL).

#### 3.3.2. LC/MS

Low-resolution LC-MS measurement was performed using an LCQ Accela Fleet (Thermo Fisher Scientific, San Jose, CA, USA) with atmospheric pressure chemical ionization (APCI) in positive ionization mode and a photodiode array detector. Luna C18(2) column, 3 μm, 150 mm × 2 mm (Phenomenex, Torrance, CA, USA), was used with a water-acetonitrile-formic acid mobile phase. Mobile phase A used 5% of acetonitrile + 0.1% of formic acid; mobile phase B used 80% of acetonitrile + 0.1% of formic acid (in vol.%). The gradient was increased from 35% of B to 40% of B during 2 min, from 40% of B to 60% of B during 10 min, from 60% B to 80% B during 1 min and from 80% B to 100% B during 1 min. The injection volume was 10 μL and the flow rate was 0.400 mL/min. The APCI capillary temperature was 275 °C, the APCI vaporizer temperature was 400 °C, the sheath gas flow was 58 L/min, the auxiliary gas flow was 10 L/min, the source voltage was 6 kV, the source current was 5 μA and the capillary voltage was 10 V.

#### 3.3.3. Liquid Chromatography–Nuclear Magnetic Resonance Spectroscopy (LC-NMR)

LC-NMR analysis followed the protocol described in our previous work [24]. Briefly, the analysis was performed using a commercial HPLC system (Dionex UltiMate 3000, Thermo Fisher Scientific) with a 4.6 × 250 mm HPLC column (Luna C18 (2), Phenomenex, 5 µm, 100 Å pore size). The concentrated acetonitrile solution (50 μL) was injected into the HPLC. The sample was separated in isocratic mode using an acetonitrile-deuterium oxide mobile phase (60% ACN-40% D_2_O) with detection at 254 nm. The flow rate was set to 0.5 mL/min. For the observation of ^1^H NMR spectra, a Varian INOVA 500 MHz spectrometer (Varian Inc., Palo Alto, CA, USA) equipped with an HCN triple resonance (60 μL active volume) micro-flow probe was used. Separation and experiments were carried out at ambient temperature (22 °C). The ^1^H NMR spectra of individual chromatographic peaks were recorded in the stop-flow mode with an accumulation of at least 256 scans (acquisition time 2 s, relaxation delay 1 s). In order to suppress residual solvent signals, the WET (water suppression enhanced through T1 effects) multiple-frequency solvent suppression method was used during the acquisition. The ^1^H-NMR spectra were referenced to the signal of acetonitrile (δ = 2.00 ppm).

The individual stilbenes were isolated using the same HPLC instrument. The concentrated solutions (50 μL) were injected repeatedly into the HPLC system, and fractions of isolated compounds were collected. Each fraction was evaporated to dryness and subsequently dissolved in an appropriate deuterated solvent (acetone-*d_6_*, methanol-*d_4_*) to enable comparison with the literature data.

#### 3.3.4. Determination of Stilbenes by NMR

The NMR experiments were conducted on a Varian INOVA 500 MHz spectrometer (Varian Inc., Palo Alto, CA, USA) operating at 499.87 MHz for ^1^H. Only the ^1^H NMR spectra of individual compounds in the corresponding deuterated solvent were recorded due to the low concentration. The identification was based on a comparison of the measured ^1^H spectra with the literature data.

The NMR data for pallidol (*Carex Lepidocarpa* Peak 1) are as follows: ^1^H NMR (from LC-NMR 60% ACN-40% D_2_O, ppm) δ: 6.97 (d, 4H, H-2a, H-6a, H-2b, H-6b, *J* = 8.5 Hz), 6.70 (d, 4H, H-3a, H-5a, H-3b, H-5b *J* = 8.4 Hz), 6.60 (d, 2H, H-14a, H-14b, *J* = 1.9 Hz), 6.12 (d, 2H, H-12a, H-12b, *J* = 2.0 Hz), 4.45 (s, 2H, H-7a, H-7b) and 3.76 (s, 2H, H-8a, H-8b), and they are in agreement with [10].

The NMR data for *trans*-ε-viniferin (*Carex Lepidocarpa* Peak 4) are as follows: ^1^H NMR (from LC-NMR 60% ACN-40% D_2_O, ppm) δ: 7.19 (d, 2H, H-2a, H-6a, *J* = 8.5 Hz) 7.15 (d, 2H, H-2b, H-6b, *J* = 8.6 Hz), 6.92 (d, 1H, H-8b, *J* = 16.4 Hz), 6.82 (d, 2H, H-3a, H-5a, *J* = 8.5 Hz), 6.74 (d, 2H, H-3b, H-5b, *J* = 8.6 Hz), 6.69 (d, 1H, H-14b, *J* = 1.8 Hz), 6.61 (d, 1H, H-7b, *J* = 16.4 Hz), 6.34 (d, 1H, H-12b, *J* = 1.8 Hz), 6.19 (d, 2H, H-10a, H-14a, *J* = 1.9 Hz), 6.17 (t, 1H, H-12a, *J* = 1.9 Hz), 5.48 (d, 1H, H-7a, *J* = 6.0 Hz) and 4.49 (d, 1H, H-8a, *J* = 6.0 Hz), and they are in accordance with our previously published data [24].

The NMR data for *cis*-miyabenol C (*Z*-miyabenol C) (*Carex Lepidocarpa* Peak 5) are as follows: ^1^H NMR (acetone-*d_6_*, ppm) δ: 7.12 (d, 2H, H-2a, H-6a, *J* = 8.3 Hz), 6.85 (d, 2H, H-3a, H-5a, *J* = 8.5 Hz), 6.72 (d, 2H, H-2c, H-6c, *J* = 8.5 Hz), 6.56 (d, 2H, H-3b, H-5b, *J* = 8.6 Hz), 6.50 (d, 2H, H-3c, H-5c, *J* = 8.5 Hz), 6.37 (d, 2H, H-2b, H-6b, *J* = 8.5 Hz), 6.33 (d, 1H, H-12c, *J* = 1.9 Hz), 6.26 (d, 1H, H-12b, *J* = 2.0 Hz), 6.24 (t, 1H, H-12a, *J* = 2.0 Hz), 6.12 (d, 1H, H-14c, *J* = 2.0 Hz), 6.09 (d, 1H, H-14b, *J* = 2.1 Hz), 5.91 (d, 2H, H-10a, H-14a, *J* = 2.1 Hz), 5.83 (d, 1H, H-8c, *J* = 12.4 Hz), 5.78 (d, 1H, H-7c, *J* = 12.4 Hz), 5.29 (d, 1H, H-7a, *J* = 3.1 Hz), 5.26 (d, 1H, H-7b, *J* = 2.6 Hz), 4.22 (d, 1H, H-8a, *J* = 3.0 Hz) and 3.87 (d, 1H, H-8b, *J* = 2.5 Hz). The NMR data for *cis*-miyabenol C (*Z*-miyabenol C) were in agreement with [25].

The NMR data for the compound from *Carex lepidocarpa* Peak 6 are as follows: ^1^H NMR (acetone-*d_6_*, ppm) δ: 7.07 (d, 2H, *J* = 8.6 Hz), 7.00 (d, 2H, *J* = 8.7 Hz), 6.99 (d, 2H, *J* = 8.6 Hz), 6.89 (d, 1H, *J* = 16.3 Hz), 6.81 (d, 2H, *J* = 8.6 Hz), 6.80 (brs, 2H), 6.74 (d, 2H, *J* = 8.6 Hz), 6.73 (d, 1H, *J* = 2.1 Hz), 6.30 (d, 1H, *J* = 2.2 Hz), 6.26 (d, 1H, *J* = 16.3 Hz), 6.22 (t, 1H, *J* = 2.3 Hz), 6.20 (d, 1H, *J* = 2.1 Hz), 5.97 (d, 1H, *J* = 2.2 Hz), 5.97 (d, 2H, *J* = 2.3 Hz), 5.30 (d, 1H, *J* = 3.6 Hz), 5.03 (d, 1H, *J* = 7.3 Hz), 4.58 (d, 1H, *J* = 7.3 Hz) and 3.70 (d, 1H, *J* = 3.6 Hz).

The NMR data for *cis*-miyabenol A (*Carex Acuta* Peak 1) are as follows: ^1^H NMR (methanol-*d_4_*, ppm) δ: 6.72 (d, 2H, H-2d, H-6d, *J* = 8.5 Hz), 6.61 (d, 2H, H-2a, H-6a, *J* = 8.6 Hz), 6.60 (d, 2H, H-3c, H-5c, *J* = 8.6 Hz), 6.51 (d, 2H, H-3a, H-5a, *J* = 8.6 Hz), 6.48 (d, 2H, H-2c, H-6c, *J* = 8.7 Hz), 6.45 (d, 2H, H-3d, H-5d, *J* = 8.6 Hz), 6.44 (d, 2H, H-2b, H-6b, *J* = 8.6 Hz), 6.40 (d, 2H, H-3b, H-5b, *J* = 8.7 Hz), 6.30 (brs, 1H, H-12d), 6.28 (d, 1H, H-12b, *J* = 2.2 Hz), 6.25 (d, 1H, H-14c, *J* = 2.2 Hz), 6.17 (d, 1H, H-12c, *J* = 2.0 Hz), 6.05 (d, 1H, H-14b, *J* = 2.2 Hz), 6.00 (d, 1H, H-14d, *J* = 2.2 Hz), 5.92 (brs, 1H, H-12a), 5.89 (d, 1H, H-7d, *J* = 12.0 Hz), 5.85 (d, 1H, H-8d, *J* = 12.0 Hz), 5.78 (brs, 2H, H-10a, H-14a), 5.40 (d, 1H, H-7c, *J* = 2.7 Hz), 5.12 (d, 1H, H-7b, *J* = 2.0 Hz), 5.02 (d, 1H, H-7a, *J* = 6.3 Hz) 4.40 (d, 1H, H-8b, *J* = 2.0 Hz) 3.98 (d, 1H, H-8a, *J* = 6.3 Hz) and 3.87 (d, 1H, H-8c, *J* = 2.8 Hz). The NMR data for *cis*-miyabenol A were in accordance with [18].

## 4. Conclusions

We focused our study on two species of sedges, *Carex acuta* and *Carex lepidocarpa*, which have been not studied yet, based on our previous screening of stilbenes in the Collection of Aquatic and Wetlands Plants in Třeboň, Institute of Botany of the Czech Academy of Sciences, Czech Republic. Pallidol, *trans-*ɛ-viniferin, *cis* and *trans*-miyabenol A and *cis*-miyabenol C were identified in the extracts. In *C. acuta*, the mixture of *cis* and *trans* miyabenol A predominates, reaching almost 5 mg/g d.m., while in *C. lepidocarpa*, the highest number of stilbenes is attributed to pallidol (up to 14.5 mg/g d.m.) and to *cis*-miyabenol C (up to 4.8 mg/g d.m.). The content of all stilbenes reaches almost 3% *w*/*w*, which is interesting for the isolation of these substances from the biomass of *C. lepidocarpa* roots and is very interesting in terms of recent literature data regarding resveratrol oligomers—specifically, the inhibition of SARS-CoV-2 entry by targeting host protease cathepsin L.

## Figures and Tables

**Table 1:** Overview of stilbenes found in different *Carex* species.

*Carex*	Part of the Plant	Stilbenes	(*m*/*z*) [Da]	Literature
*C. appessa*	seeds	virgatanol	[M + H]^+^ 471	[10]
		resveratrol diglucoside	[M + H]^+^ 553	
		piceatannol	[M + H]^+^ 245	
		ε-viniferin	[M + H]^+^ 455	
*C. buchananii*	roots	kobophenol A		[11]
*C. capillacea*	roots	(*E*)-miyabenol A		[11]
*C. ciliato-marginata*		(+)-α-viniferin	[M + H]^+^ 679	[12]
		pallidol	[M + H]^+^ 455	
		kobophenol A	[M + H]^+^ 925	
*C. cuprina*	roots	carexinol A	[M + H]^+^ 941	[11]
		kobophenol A	[M + H]^+^ 925	
*C. distachya*	roots	pallidol diglucoside		[13]
*C. fedia*	roots, rhizomes	ε-viniferin		[14]
		miyabenol A		
		miyabenol B		
		miyabenol C		
*C. foliosissima*		(+)-α-viniferin	[M + H]^+^ 679	[12]
		pallidol	[M + H]^+^ 455	
		kobophenol A	[M + H]^+^ 925	
*C. folliculata*	seeds	pallidol		[15]
		kobophenol A		
*C. folliculata*	seeds	pallidol		[7]
		kobophenol A		
*C. glauca*	roots	(*E*)-miyabenol C	[M + H]^+^ 681	[14]
		(+)-α-viniferin	[M + H]^+^ 679	
*C. gynandra*	aerial part	pallidol		[5]
		α-viniferin		
		*trans*-miyabenol C		
		kobophenol B		
*C. hirta*	roots	resveratrol-diglucoside	[M + H]^+^ 553	[11]
		(*E*)-miyabenol A	[M + H]^+^ 907	
*C. humilis*	roots	α-viniferin		[16]
*C. kobomugi*	roots	kobophenol A		[17]
*C. morrowii*		(+)-α-viniferin	[M + H]^+^ 679	[12]
		pallidol	[M + H]^+^ 455	
		kobophenol A	[M + H]^+^ 925	
*C. multufolia*		(+)-α-viniferin	[M + H]^+^ 679	[12]
		pallidol	[M + H]^+^ 455	
		kobophenol A	[M + H]^+^ 925	
*C. pendula*	seeds	*cis*-miyabenol A	[M + H]^+^ 907	[18]
		*cis*-miyabenol C	[M + H]^+^ 681	
		kobophenol B	[M + H]^+^ 905	
*C. pumita*	roots, rhizomes	(−)-ε-viniferin		[19]
		miyabenols A, C		
*C. vulpinoidea*	seeds	vulpinoideol A	[M + Na]^+^ 481	[20]
		vulpinoideol B	[M + Na]^+^ 511	

**Table 2:** Stilbenes in the roots of *Carex acuta* and *Carex lepidocarpa* (chromatogram in Figure 2.).

Stilbenes	Retention Time [min]	*m*/*z* [M + H]^+^ [Da]
Pallidol (peak 1)	4.02	455
*trans*-resveratrol (peak 2)	5.51	229
Mixture *cis+trans* miyabenol A (peak 3)	6.99	907
*trans-*ɛ-viniferin (peak 4)	7.13	455
*Z*-miyabenol C (peak 5)	7.45	681
Trimer (peak 6)	7.78	681

**Table 3:** Content of stilbenes in the roots of *Carex acuta* and *Carex lepidocarpa* (µg/g dry matter); R.t. (drying at room temperature), Lyof. (drying by lyophilization).

Stilbenes/Sample Drying	*C. acuta* 2016 R. t.	*C. acuta* 2017 Lyof.	*C. acuta* 2017 R. t.	*C. lepidocarpa* 2016 R. t.	*C. lepidocarpa* 2017 Lyof.
Pallidol (peak 1)	308 ± 54	270 ± 16	194 ± 28	14,478 ± 976	13,087 ± 1755
*trans*-resveratrol (peak 2)	88 ± 12	78 ± 3	84 ± 10	70 ± 6	66 ± 11
Mixture *cis+trans* Miyabenol A (peak 3)	3641 ± 505	4572 ± 586	2603 ± 227	1890 ± 273	847 ± 14
*trans-*ɛ-viniferin (peak 4)	n.d.	n.d.	n.d.	5035 ± 294	3777 ± 332
*cis*-miyabenol C (peak 5)	263 ± 5	487 ± 55	342 ± 32	4805 ± 279	3209 ± 185
Trimer (peak 6)	174 ± 18	102 ± 7	96 ± 9	5060 ± 147	4065 ± 267

n.d.: not detected. LOD for *trans*-ε-viniferin 0.089 μg/mL, LOQ 0.298 μg/mL.

## Data Availability

The data used to support the findings of this study can be made available by the corresponding author upon request.
